# Precision health for breast cancer metastasis: biomaterial scaffolds as an engineered metastatic niche to define, study, and monitor metastatic progression

**DOI:** 10.18632/oncoscience.493

**Published:** 2019-12-23

**Authors:** Grace G. Bushnell, Max S. Wicha, Jacqueline S. Jeruss, Lonnie D. Shea

**Affiliations:** ^1^Department of Internal Medicine, University of Michigan, Ann Arbor, MI, USA; ^2^Department of Biomedical Engineering, University of Michigan, Ann Arbor, MI, USA; ^3^Department of Surgery, University of Michigan, Ann Arbor, MI, USA; ^4^Department of Chemical Engineering, University of Michigan, Ann Arbor, MI, USA

**Keywords:** cancer metastasis, biomaterial implant, breast cancer, Hi-C, RNAseq

## Abstract

Metastasis represents the greatest challenge to treatment of cancer patients. Biomaterial scaffolds that recruit tumor cells to a defined site in vivo are an emerging platform for the diagnosis, treatment, and study of metastasis. Recruitment of immune cells and metastatic tumor cells to a defined location provides a precision health platform to assess current clinical cancer biomarkers in a metastatic setting, and to define the next generation of biomarkers. These platforms represent an opportunity to create a molecular staging of metastasis that could aid in both the early diagnosis and treatment of metastasis.

Metastasis represents the most common cause of death for patients with breast cancer. As a result of limitations of current technologies to detect early metastasis, they are usually not detected until they produce symptoms or are detected on scans. An emerging pre-clinical technology to detect early metastasis is a biomaterial scaffold that functions as a synthetic metastatic niche to recruit metastatic tumor cells [1]. These platforms robustly recruit tumor cells across several mouse models of metastasis [1]. Tumor cells recruited to the synthetic niche are representative of metastasis to other organs, and reflect an aggressive population; similar in metastatic ability, behavior *in vitro*, and transcriptome to breast cancer cells that spontaneously metastasized to the lung [2]. These findings suggest that tumor cells recruited to a synthetic niche could provide a surrogate for tumor cells in occult or relatively inaccessible locations. Additionally, the phenotype of recruited tumor cells may be reflective of disease biology and aggressiveness. This platform technology provides a precision health platform to access clinical biomarkers expressed in the metastatic niche (Figure1). Biomarkers are analyzed in the primary tumor for identification of subtype or proliferative index, to determine a relative risk of recurrence and treatment plan. However, for most cancers, metastases, not the primary tumor, are responsible for mortality. Tumor cell phenotypes may differ between the primary tumor and metastatic sites [3], and thus utilization of this synthetic niche provides an opportunity to gain insight into tumor biology and subtype at an accessible metastatic site. Furthermore, the synthetic niche captures early metastatic cells [4], prior to colonization of other distal tissues, and could provide a molecular staging of metastasis that guides the rational choice of therapy based on the unique biology of emerging metastases, prior to organ compromise. The synthetic niche recruits tumor cells in part through immune cell function [1] whose analysis (e.g., PD1 and PD-L1) can inform selection of immunotherapies. Emerging evidence suggests that PD1 and PD-L1 expression are differentially expressed in primary tumor and metastases [5]. Given that tumor subtype may differ between primary and metastatic disease, the differential expression of immune checkpoints would be unsurprising. Patients who have PD-L1 positive primary tumors, yet who have PD-L1 negative metastases, would benefit from receiving an appropriately targeted therapy while avoiding treatment delays from exposure to ineffective and potentially toxic agents. Additionally, the synthetic niche platform has potential benefits beyond enumeration of circulating tumor cells (CTCs) or disseminated tumor cells (DTCs), which has been performed as part of clinical research for more than two decades yet has not been routinely applied in clinical decision making. The presence of an easily accessible metastatic site would facilitate analysis of cells proven to be capable of metastasis [2], which distinguish them from the majority of CTCs and DTCs. Additionally, the analysis of CTCs and DTCs does not account for the microenvironment that tumor cells occupy and co-opt during the metastatic cascade. To this end, the synthetic niche recapitulates many cellular and acellular elements of the natural metastatic niche, and reflects the cellular alterations that occur systemically [6]. Collectively, the synthetic niche provides a platform for analysis of metastatic tumor cells, and the environment that influences their function and phenotype. The synthetic niche also provides a platform to discover new biomarkers for treatments based on staging of metastasis. The future of personalized medicine will require an extension beyond the current biomarkers which have been developed based on the primary tumor. New biomarkers for metastasis are necessary as metastases are not typically detected until relatively late. In addition to detecting early metastases, the synthetic niche captures both tumor and niche stromal cells, which allows analysis of the complex interplay between tumor cells and their microenvironment from the initiation of the metastatic niche and throughout niche maturation. In contrast to vital organs, the synthetic niche can be repeatedly sampled with core biopsy without significant patient risk, and this longitudinal monitoring could be useful in both pre-clinical models and human clinical trials as a key feature of discovery. Gene expression and phenotypic analysis of the microenvironment, including myeloid derived suppressor cells (MDSCs), macrophages, endothelial cells, and fibroblasts, would be expected to correlate with disease progression [1]. Paracrine communication originating from stromal cells can influence epigenetic programming of the tumor cells, influencing properties such as stemness, dormancy, and resistance [7,8]. Identifying the mechanisms driving niche maturation and metastatic progression would serve as staging biomarkers and may also function as targets for therapeutic intervention aimed at disrupting the microenvironmental support of metastatic tumor cells. Tumor cells recruited to the synthetic niche have characteristics similar to the native metastatic sites [2]. Interestingly, these tumor cells can be expanded [2] which could enable their use for drug screening platforms, or mutations and neoantigens may be identified that could be the basis of targeted therapeutics, or cancer vaccines, respectively [9]. This analysis of the microenvironment and tumor cells could provide the basis of a metastatic staging of disease, informing metastasis-specific therapies, and subsequent analysis of the scaffold could potentially determine sensitivity or resistance to the therapy. In conclusion, a synthetic metastatic niche provides a pre-defined site that enables analysis of metastases from initiation to progression that is not possible due to the occult, focal, and stochastic nature of metastatic sites in vital organs. The scaffold tool is an enabling precision medicine technology to elucidate the biology and paracrine signaling that occurs at metastatic sites, which can be exploited for monitoring disease progression (or regression) and development of therapies.

**Figure 1:**
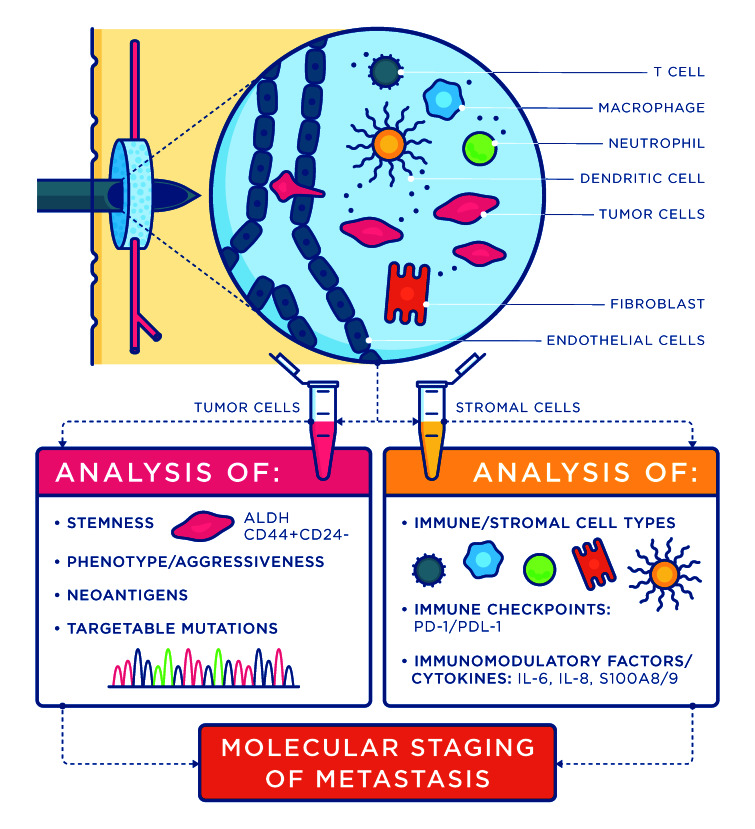
Engineered metastatic niche as a site to develop a molecular staging of metastasis. Biomaterial scaffolds serving as an engineered metastatic niche provide an opportunity to capture both tumor cells and metastasis associated stromal and immune cells. These cells can be analyzed using current clinical markers and may also be used to develop the next generation of metastasis biomarkers. Taken together, the engineered metastatic niche can provide a molecular staging of metastasis.
